# TGF-β1-induced miR-503 controls cell growth and apoptosis by targeting PDCD4 in glioblastoma cells

**DOI:** 10.1038/s41598-017-11885-8

**Published:** 2017-09-14

**Authors:** Pin Guo, Yanan Yu, Huanting Li, Daoxiang Zhang, Anjing Gong, Shifang Li, Wei Liu, Lei Cheng, Yongming Qiu, Weicheng Yao, Luo Li, Yugong Feng

**Affiliations:** 1 Department of Neurosurgery, the Affiliated Hospital of Qingdao University, Qingdao, China; 2 Department of Gastroenterology, the Affiliated Hospital of Qingdao University, Qingdao, China; 3 Division of Oncology, Department of Internal Medicine, Washington University School of Medicine, Saint Louis, MO 63110 USA; 4 Department of Neurosurgery, South Campus, Renji Hospital, Shanghai Jiao Tong University School of Medicine, Shanghai, China; 5 Department of Neurology, Qingdao Municipal Hospital, Qingdao University, Qingdao, China

**Keywords:** Targeted therapies, Oncogenes

## Abstract

Aberrant expression of microRNAs hae been shown to be closely associated with glioblastoma cell proliferation, apoptosis and drug resistance. However, mechanisms underlying the role of mcroRNAs in glioblastoma cell growth and apoptosis are not fully understood. In this study, we report that miR-503 is overexpressed in glioblastoma tissue compared with normal human brain tissue. Mechanistically, miR-503 can be induced by TGF-β1 at the transcriptional level by binding the smad2/3 binding elements in the promoter. Ectopic overexpression of miR-503 promotes cell growth and inhibits apoptosis by targeting PDCD4. In contrast, inhibition of miR-503 reduces cell growth. Furthermore, miR-503 inhibitor augments the growth inhibitory effect of temozolomide in glioblastoma cells. These results establish miR-503 as a promising molecular target for glioblastoma therapy.

## Introduction

Glioblastoma, which accounts for approximately 80% of primary malignant brain tumors, is among the most prognostically discouraging adult neoplasias^1^. The annual incidence of glioblastoma is about 3–8 per 10 million people^2^. The median survival of glioblastoma patients is less than one year from diagnosis, which is lower than that for patients with any other kind of tumor^3^. Current therapies for glioblastoma, including radiotherapy, surgery and chemotherapy, have not been successful due to the highly aggressive nature of the tumor^1, 2^. Therefore, there is an urgent demand to understand the molecular mechanisms controlling glioblastoma progression and manifestation.

MicroRNAs (miRNAs) are a class of 18–22 bp non-coding RNAs that play key roles in cell proliferation, apoptosis and differentiation^4^. MicroRNAs can act as both oncogenes and tumor suppressors by negatively regulating mRNA through either translational repression or mRNA degradation^5^. In recent years, the aberrant expression of many microRNAs has been reported to be associated with progression of various cancers. These aberrantly expressed microRNAs have been identified as novel potential targets for cancer therapy^6^. Therefore, exploring the role and the mechanism of microRNAs dysregulation in cancer is essential for diagnosis and therapy.

Aberrant expression of miR-503 has been shown in several types of cancers and appears to be significantly associated with clinical outcome in patients. For example, downregulation of microRNA-503 expression level predicates advanced cytological features and poor prognosis in patients with non-small cell lung cancer^7^. In prostate cancer, microRNA-503 can directly regulate RNF31 and thus suppress tumor cell proliferation and metastasis^8^. Analysis of microRNA expression profiling shows that microRNA-503 regulates metastatic function in hepatocellular cancer cells^9^. However, microRNA-503 was found to be upregulated in esophageal cancer tissues compared to adjacent normal tissues and to promote tumor progression^10^. Feinmesser *et al*. reported that upregulation of miR-503 was the best single discriminator of malignancy^11^. On the basis of these previous reports, downregulation or upregulation is cancer type-specific. In glioblastoma, the role of miR-503 remains largely unknown.

In this report, we found that miR-503 is overexpressed in human glioblastoma tissues compared with normal brain tissues, and that TGF-β1 can induce miR-503 expression at the transcriptional level by binding the promoter. Our biological function assay showed that miR-503 enhances cell proliferation and inhibits apoptosis by targeting PDCD4. Furthermore, miR-503 inhibitor augments the growth-inhibitory effect of temozolomide in glioblastoma cells. These results establish miR-503 as a promising molecular target for glioblastoma therapy.

## Results

### miR-503 is up-regulated in glioblastoma

**Figure 1:**
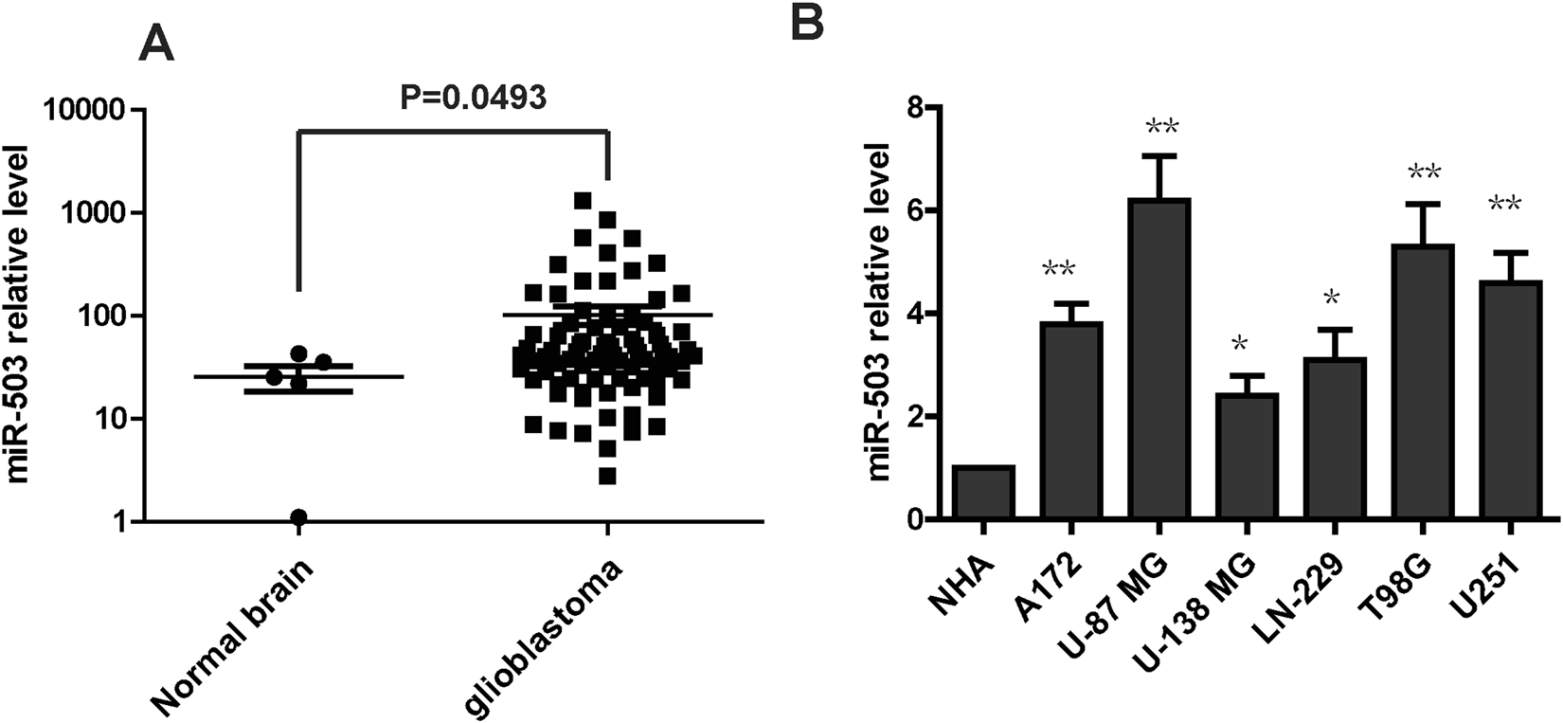
miR-503 is up-regulated in glioblastoma. (**A**) miR-503 expression was analyzed from the microRNA profiling in glioblastoma tissues and normal brain tissues. (**B**) The level of miR-503 was individually analyzed using quantitative PCR analysis insix glioblastoma cell lines (U251, A172, LN-229, T98G, U87MG and U-138MG) and in normal human astrocytes (NHAs).

To examine the expression level of miR-503 in human glioblastoma, we analyzed the miRNA’s expression profile from a previously published dataset (Gene expression omnibus accession GSE25631). The result showed that miR-503 is significantly upregulated in glioblastoma tissue compared with normal brain tissue (p < 0.001) (Fig. 1A). To further confirm the expression level of miR-503 in glioblastoma, we performed qRT-PCR to detect the level of miR-503 in glioblastoma cell lines. The result showed that the miR-503 level is higher in the six glioblastoma cell lines (U251, A172, LN-229, T98G, U87MG and U-138MG) than in normal human astrocytes (NHAs) (Fig. 1B). In conclusion, miR-503 is overexpressed in glioblastoma.

### TGF-β1 induces the expression of miR-503 in glioblastoma cells

**Figure 2:**
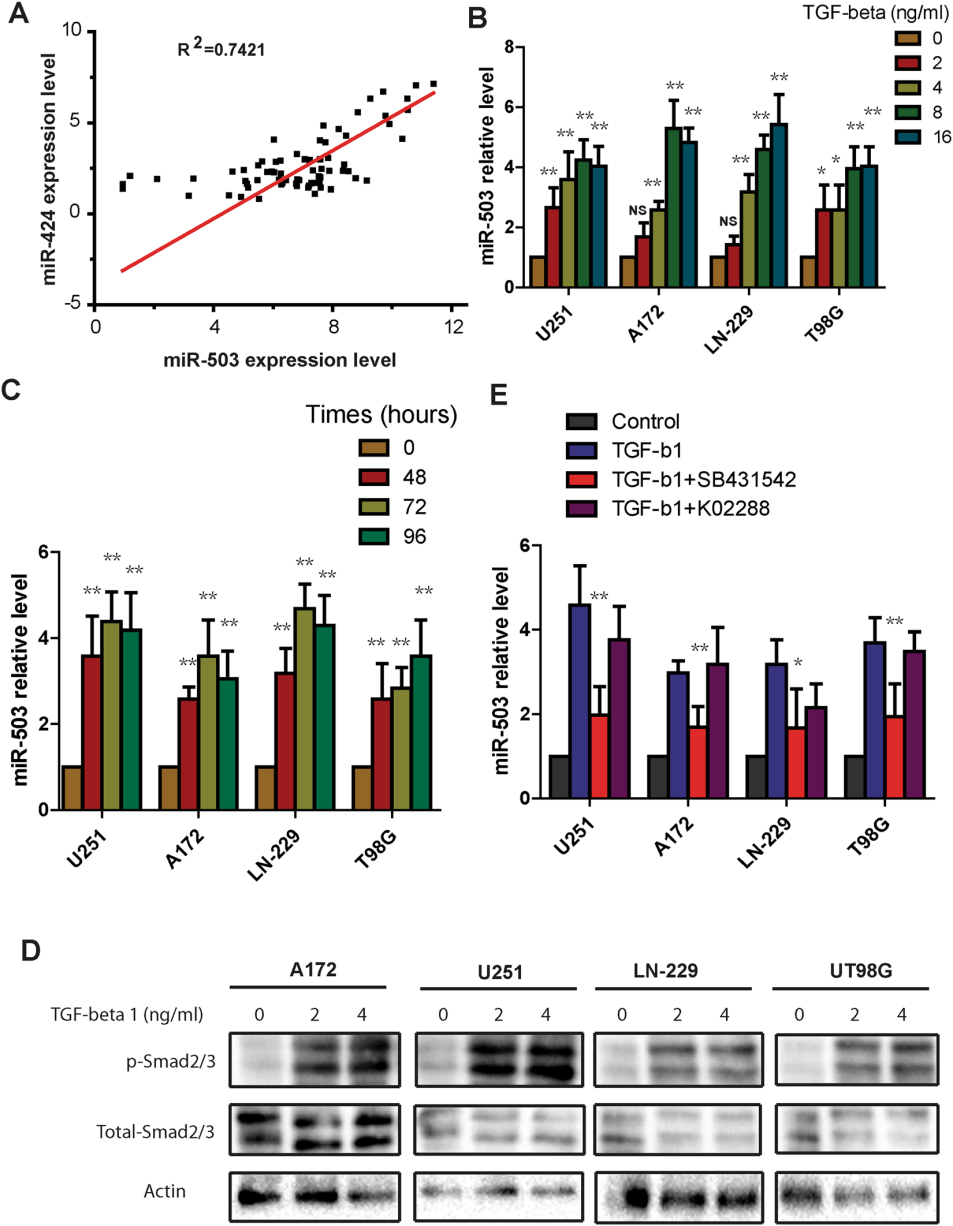
TGF-β1 induces the expression of miR-503 in glioblastoma cells. (**A**) Correlation analysis of miR-503 level and miR-424 level in the NCI 60 cancer cell panel using Pearson correlation (R^2^ = 0.7421). (**B**) Quantitative PCR analysis of miR-503 expression in U251, A172, LN-229 and T98G cells treated with TGF-β1 at different doses for 48 h. (**C**) Quantitative PCR analysis of miR-503 expression in U251, A172, LN-229 and T98G cells treated with 8ng/ml TGF-β1 at different time points. (**D**) Western blot analysis showing the phosphorylation of Smad2/3 in U251, A172,LN-229 and T98G cells treated with TGF-β1 at different doses for 30 min. (**E**) Quantitative PCR analysis of miR-503 expression in U251, A172, LN-229 and T98G cells treated with TGF-β1 alone or with SB-431542 or K02288 for 48 h.

Previous studies have shown that miR-503 is dysregulated in several types of cancer, however, the mechanism underlying the regulation of miR-503 in cancer remains unknown. Jongmin Kim *et al*. reported that miR-503 and miR-424, which are separated by 250 bp on the X chromosome, are transcribed as a single transcript. To explore whether there is a relationship between the levels of miR-503 level and miR-424, we analyzed the global microRNA expression of the published NCI-60 cancer cell panel. We found that miR-503 and miR-424 levels have a strong correlation (R2 = 0.7421) (Fig. 2A), which indicates that miR-503 and miR-424 may be governed by the same mechanism. Zhang *et al*. reported that miR-424 can be induced by TGF-β1 in normal fibroblasts. To explore whether TGF-β1 induces miR-503 expression, we treated glioblastoma cells with different TGF- β1 doses, ranging from 2ng/ml to 16ng/ml, and then performed qRT-PCR to determine miR-503 levels. The result showed that expression of mature miR-503 increased with the TGF-β1 dose increase (Fig. 2B). To further confirm the effect of TGF-β1 on miR-503 expression, TGF-β1 was administered to glioblastoma cells at final concentration of 8 ng/ml and qRT-PCR was performed at different time points (0, 48, 72, and 96 hours). qRT-PCR showed that miR-503 expression was enhanced under TGF-β1 treatment, and was highest at 72 hours in three glioblastoma cell lines and at 96 hours in T98G cells (Fig. 2C). As smad2/3 is the key mediator of the canonical TGF-β1 pathway, we explored whether endogenous need of TGF-β1 pathway for the miR-503 regulation in glioblastoma cells. To investigate this, we examined the response of glioblastoma cells to TGF-β1 signaling. Western blot results showed the phosphorylation of smad2/3 is markedly enhanced in four glioblastoma cell lines under TGF-β1 treatment for 30 minutes (Fig. 2D). To further confirm this, we detected the PAI-1 and Smad 7 mRNA level in glioblastoma cells treated with TGF-β1, the data showed that PAI-1 mRNA was increased significantly in four cells treated with TGFb1 for 48 h, however Smad7 mRNA was not enhanced as highly and significantly as PAI-1 mRNA (Fig. 1S). Next we treated the cells with TGF-β1 alone or with K02288 or SB-431542, which are selective inhibitors involved in BMP signaling and TGF-β signaling, respectively. qRT-PCR showed that miR-503 expression was suppressed by SB-431542 inhibitor, but not by K02288 inhibitor under TGF-β1 treatment. (Fig. 2E). Taken together, these data indicate that TGF-β1 regulates the expression of miR-503 in glioblastoma via the smad2/3 pathway.

### TGF-β1 induces the expression of miR-503 at the level of transcription

**Figure 3:**
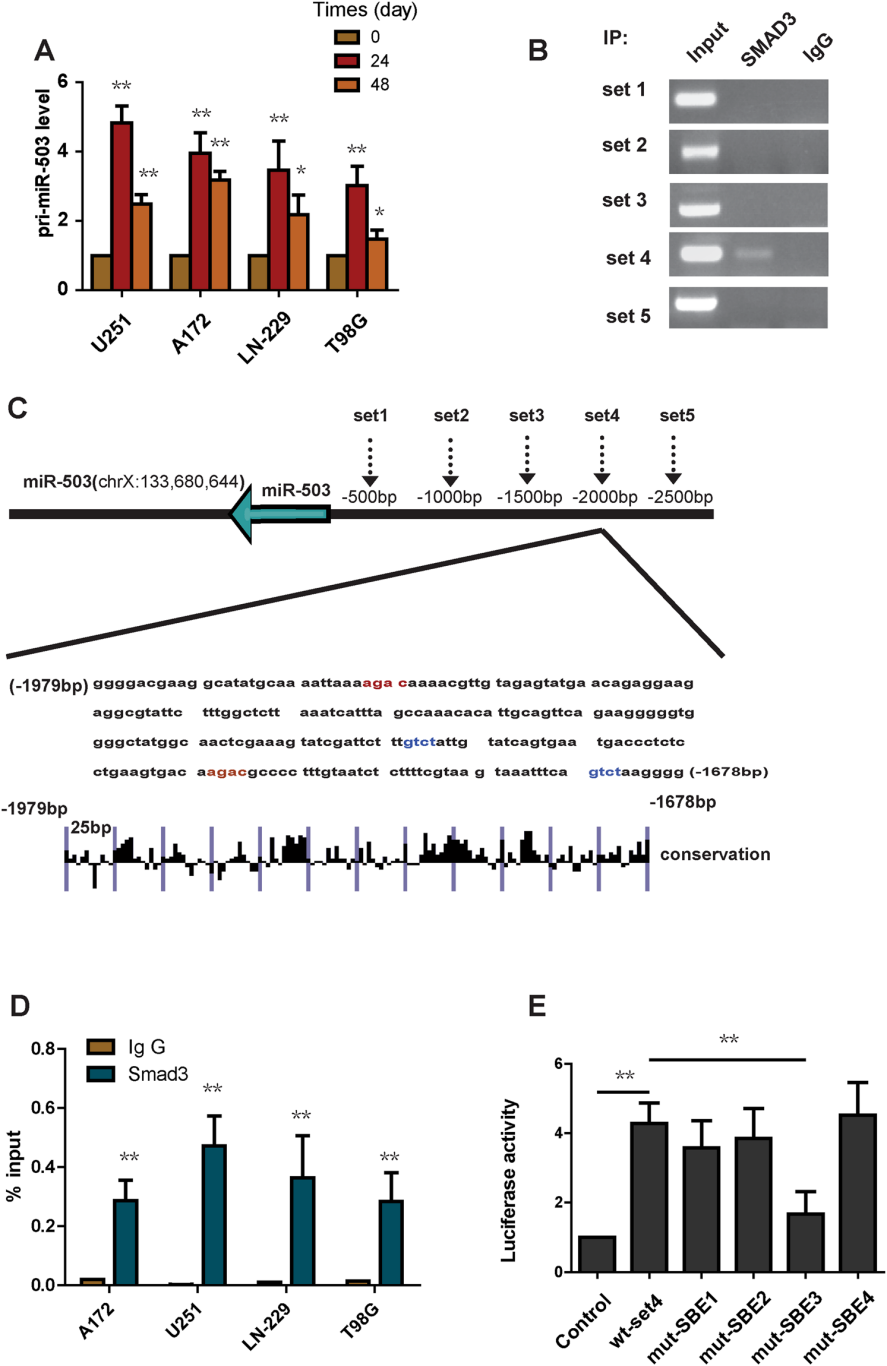
TGF-β1 induces the expression of miR-503 at the transcriptional level. (**A**) Pri-miR-503 expression was analyzed using quantitative PCR in U251, A172, LN-229 and T98G cells treated with TGF-β1. (**B**) ChIP-PCR amplification products using primers against the miR-503 promoter region were separated by agarose gel electrophoresis. (**C**) Evolutionary conservation of the set4 region in miR-503 promoter was shown. (**D**) qChIP analysis showing the enrichment of Smad2/3 in set 4 in U251, A172, LN-229 and T98G cells. (**E**) The luciferase reporter assay showing that mut-SBE3 significantly inhibits the increased luciferase activity induced by TGF-β1.

TGF-β1 signaling modulates downstream gene expression at the transcriptional level. This is mediated through the transcription factors smad2, smad3 and smad4^12, 13^. To explore whether TGF-β1 stimulation could induce miR-503 expression at the transcriptional level, we detected the pri-miR-503 level in glioblastoma cells under TGF-β1 treatment. qRT-PCR showed that TGF-β1 administration strongly induced pri-miR-503 expression (Fig. 3A). To further identify how TGF-β1 regulates miR-503 expression, we analyzed the 2500 bp region upstream of pri-miR-503 using five pairs of primers to create a library of five 500 bp regions spanning the miR-503 promoter (Fig. 3C). We tested each of these regions for association with SMADs. We performed ChIP experiments for smad2/3. Enriched DNA from immunoprecipitates was used for PCR and RT-PCR. Only the set 4 region of the promoter was found to be associated with enrichment of smad2/3 (Fig. 3B). No enrichment was detected with an isotype-matched IgG antibody (Fig. 3D). In the set 4 region, we found 4 copies of the sequence GTCT or AGAC (smad-binding elements, SBEs) (Fig. 3C), which can be bound by the smad3/smad4 complex. To further explore whether smad2/3 binds the SBEs, we cloned the set 4 region into the pGL3 promoter and mutated each SBE individually. HEK293T cells were transfected with the control plasmid, wild type set 4 region plasmid or mutant plasmid. In the luciferase reporter assay, wild-type set 4 region plasmid significantly enhanced the luciferase activity (Fig. 3E). A mutation in SBE 3 led to a significant decrease in luciferase activity, however, mutation in SBE1, SBE2, SBE4 showed no decreased luciferase activity compared with the wild-type set 4 region plasmid (Fig. 3E). We also found that the SBE4 is highly conserved in the promoter region (Fig. 3C). In conclusion, Smad2/3 binds to the SBE3 in the set 4 region of the pri-miR-503 promoter and directly activates its transcription.

### miR-503 suppresses PDCD4 expression

**Figure 4:**
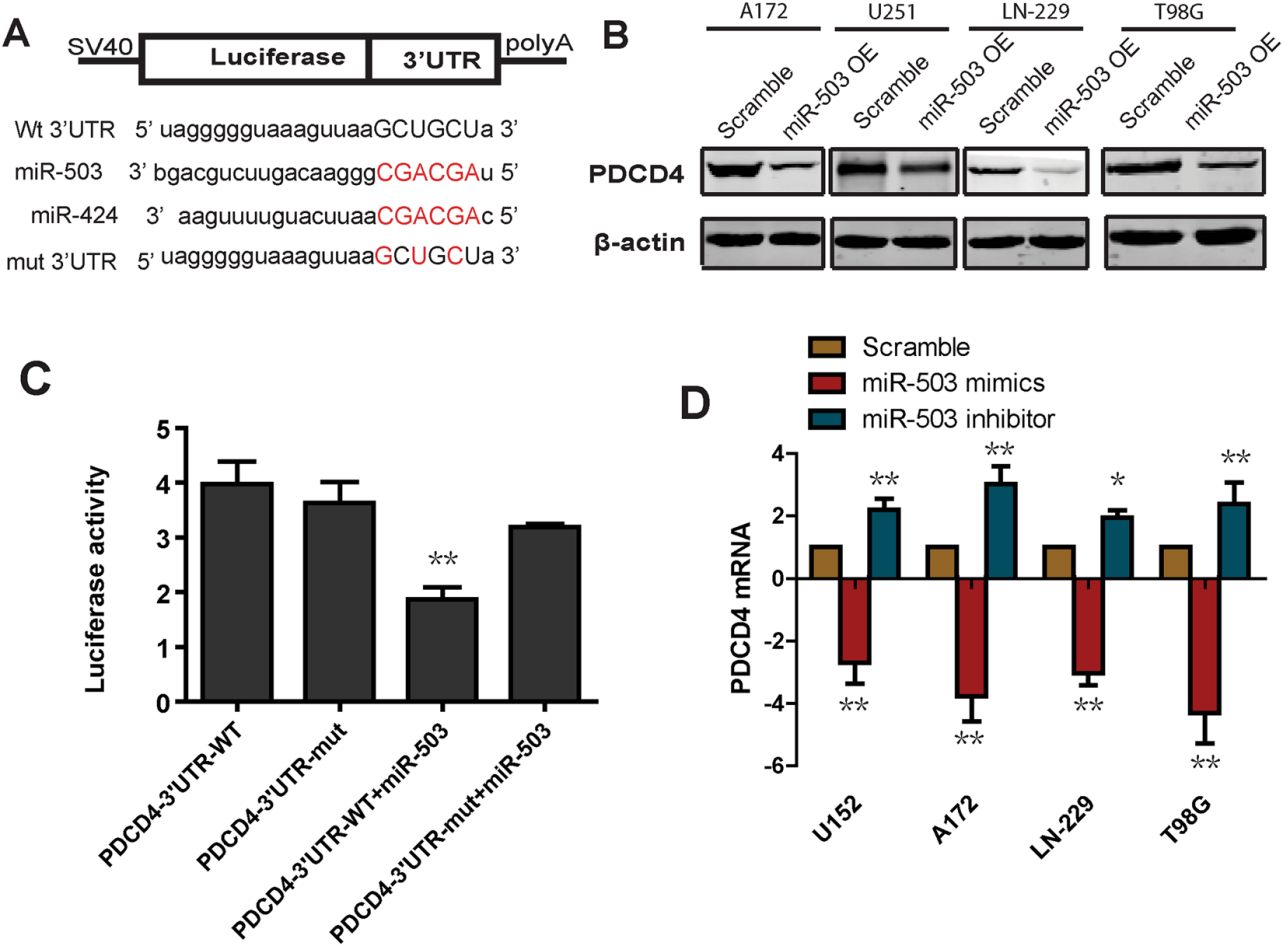
miR-503 suppresses PDCD4 expression. (**A**) The 3′ UTR sequence of PDCD4 is predicted as a potential binding site for miR-503. The red nucleotides are mutated to their complementary nucleotides. The blue nucleotides are the seed region of miR-424 and miR-503. (**B**) Western blot results showing the level of PDCD4 protein in U251, A172 and LN-229 cells transfected with miR-503 mimics. (**C**) Luciferase reporter assay showing the luciferase activity in cells transfected with the wild type 3′ UTR or the mutant 3′ UTR with miR-503 mimics. (**D**) Quantitative PCR analysis of the PDCD4 mRNA level in U251, A172, LN-229 and T98G cells transfected with miR-503 inhibitor or miR-503 mimics.

To further detect the mechanism by which miR-503 increases glioblastoma cell growth and suppresses apoptosis, we compared the seed region sequences of miR-503 and miR-424. These two miRNAs share substantial sequence identity in their seed region (Fig. 4A)^14^. miR-424 was reported to promote tumor resistance to apoptosis by targeting the expression of PDCD4,, which is a well-established tumor suppressor, known to regulate cell growth and apoptosis^15^. On the basis of these findings, we hypothesized that PDCD4 is a potential target of miR-503. We detected the level of PDCD4 protein using western blot in cells overexpressing miR-503. The data showed that overexpression of miR-503 dramatically decreased the level of PDCD4 protein (Fig. 4B). Moreover, we analyzed the level of PDCD4 mRNA using qRT-PCR in glioblastoma cells transfected with miR-503 inhibitor or mimics. The data showed that miR-503 inhibitor increased the level of *PDCD4* mRNA. In contrast, miR-503 mimics reduced the PDCD4 mRNA level (Fig. 4D). Furthermore, to confirm whether PDCD4 is indeed the target of miR-503, we cloned the 3′ UTR of PDCD4 into the dual-luciferase UTR vector and performed the luciferase reporter assay (Fig. 4C). The data showed that luciferase activity was significantly decreased in cells cotransfected with miR-503 mimics and 3′ UTR-wild-type, but not in cells cotransfected with miR-503 mimics and 3′ UTR-mutant. Taken together, these data suggest that miR-503 downregulates the expression of PDCD4.

### miR-503 increases glioblastoma cell proliferation and inhibits cell apoptosis

**Figure 5:**
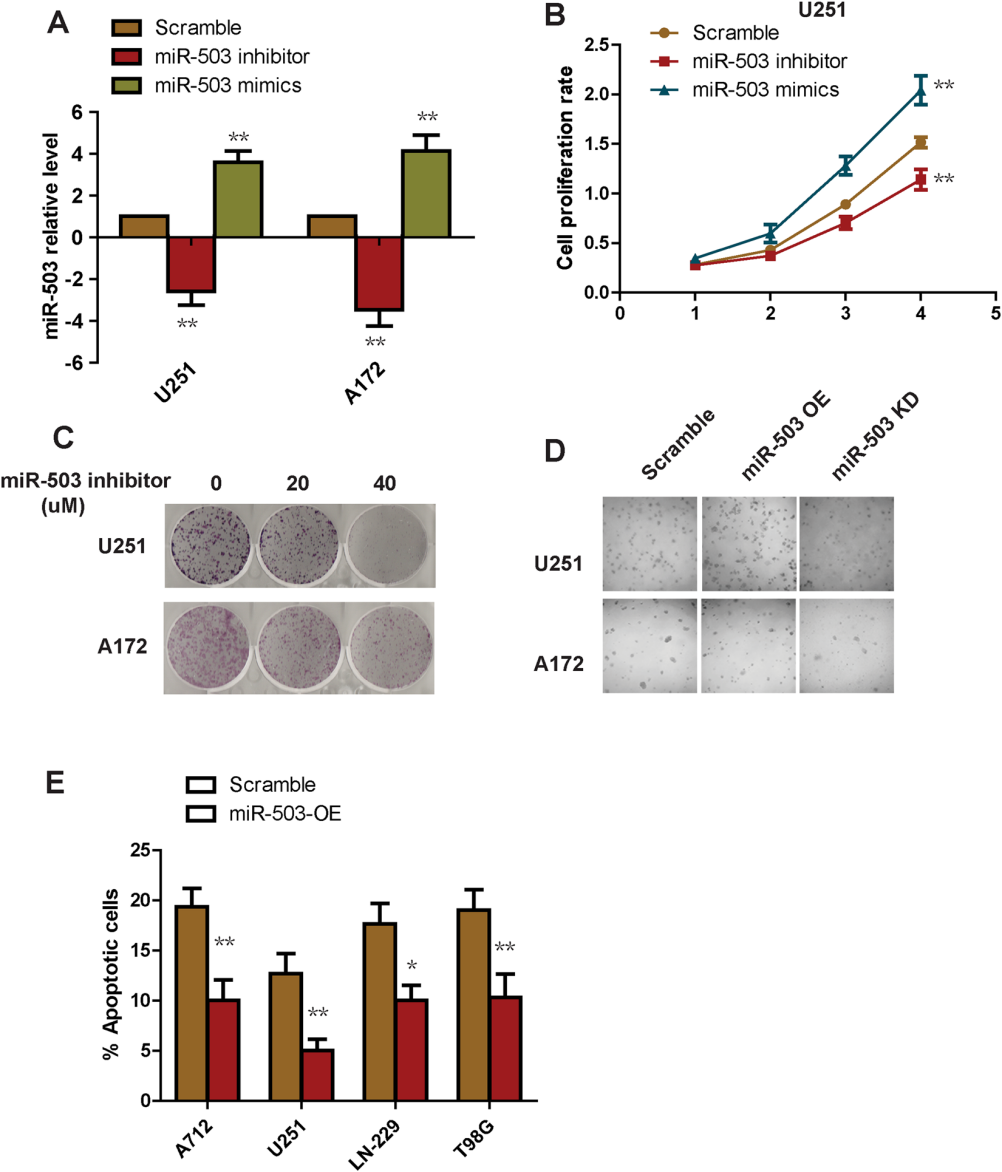
miR-503 increases glioblastoma cell proliferation and inhibits cell apoptosis. (**A**) The knockdown or overexpression efficacy of miR-503 was analyzed using quantitative PCR in U251 and A172 transfected with miR-503 inhibitor or miR-503 mimics. (**B**) The cell proliferation rate was measured with the Alarma Blue assay in cells transfected with miR-503 inhibitor or miR-503 mimics. (**C**) Colony formation assay of cells transfected with miR-503 inhibitors. (**D**) Anchorage growth of cells transfected with miR-503 mimics in soft agar. (**E**) Flow cytometry analysis of the annexin-V positive populations of cells treated with miR-503 mimics..

To examine the biological role of miR-503 in glioblastoma cells, we transfected glioblastoma cells with miR-503 inhibitor or mimic to knockdown or overexpress the endogenous miR-503, respectively. We used qRT-PCR to demonstrate that miR-503 was indeed dramatically decreased or enhanced as expected (Fig. 5A). We seeded the miR-503 knockdown or overexpression cells in 96 well plates and cultured them to different time points for the Alarma Blue cell growth assay. The results showed that overexpression of miR-503 enhanced cell proliferation, and in contrast, knockdown of miR-503 suppressed glioblastoma cell growth (Fig. 5B). We next tested the effect of miR-503 inhibitor on colony formation *in vitro*. We found that miR-503 inhibitor strongly repressed colony formation in a dose dependent manner (Fig. 5C). To further confirm these findings, we then performed the soft agar assay, which is widely accepted as the most stringent assay for transformed growth. The result showed that overexpression of miR-503 enhanced anchorage-independent growth, as expected, and knockdown of miR-503 suppressed anchorage-independent growth (Fig. 5D), indicating an essential role of miR-503 in cell growth. We next sought to explore the role of miR-503 in apoptosis. We stained cells overexpressing miR-503 with annexin V and PI for flow cytometry analysis. The data showed a lower percentage of annexin V positive cells in the miR-503 overexpression group than in the scramble cells (Fig. 5E), suggesting miR-503 overexpression suppresses glioblastoma cell apoptosis.

### miR-503 inhibitor and temozolomide inhibit glioblastoma cell growth alone and in combination

**Figure 6:**
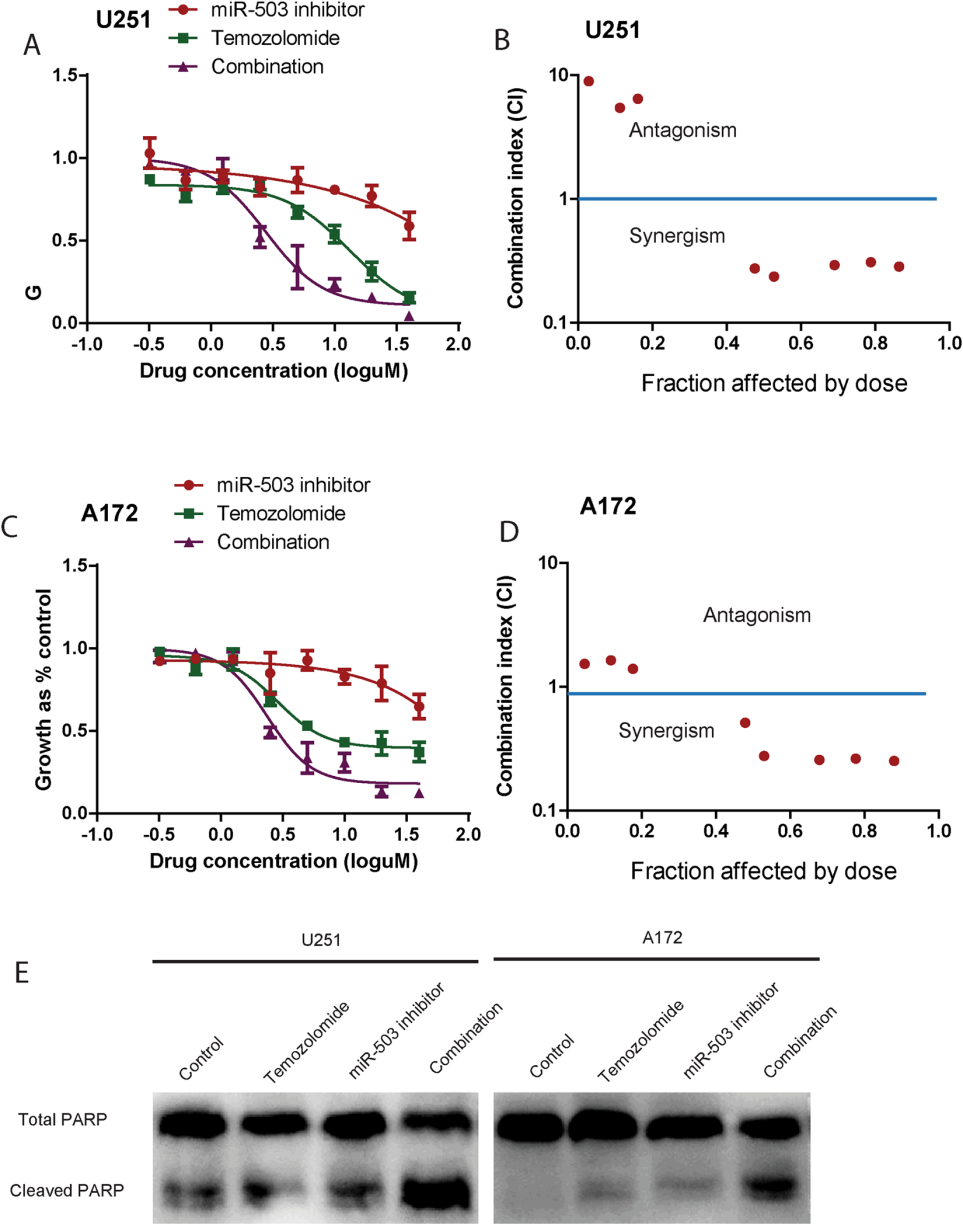
Growth inhibition induced by miR-503 inhibitor and temozolomide, alone and in combination. (**A**) and (**C)**. U251 and A172 cells were treated with various combinations of miR-503 inhibitor and temozolomide for 72 h. The growth-inhibitory effect was measured by the Alarma Blue assay. (**B**) and (**D**) Median effect analysis conducted using Calcusyn software showing interaction between miR-503 inhibitor and temozolomide in U251 and A172 cells. The combination effect was plotted as CI against the fraction affected. When the CI value is <1, the interaction between two drugs is considered synergistic, when CI = 1, the interaction is additive, and when CI > 1, the interaction is antagonistic. (**E**) The total PARP and cleaved PARP were detected using western blot analysis in U215 and A172 cells, which were transfect with a scrambled oligo or miR-503 inhibitor. The final concentration of temozolomide was 10 µM.

To translate the above findings, we next examined the effect of combined miR-503 inhibitor and the first-line glioblastoma multiforme treatment, temozolomide^16^, on glioblastoma cells over 72 h. The miR-503 inhibitor and temozolomide were used alone at 0.31 uM, 0.62 uM, 1.25 uM, 2.5 uM, 5 uM, 10 uM, 20 uM, 40 uM, and in combination at equipotent concentrations at the same ratios. The Alarma Blue assay showed that these two compounds were more growth inhibitory in combination than either compound alone (Fig. 6A,C). We then performed median effect analysis to examine whether the higher growth inhibitory rate of the combinations reflected an additive or a synergistic effect. The data showed that all combinations of miR-503 inhibitor and temozolomide were synergistic at the combined concentrations of 5 uM, 10 uM, 20 uM, and 40 uM. These data showed that miR-503 inhibitor augmented the growth inhibitory effect of temozolomide (Fig. 6B,D). The western blot showed that miR-503 inhibitor augmented the level of PARP cleavage, an indicator of apoptosis, when combined with temozolomide. Taken together, these results provide a potential preclinical rationale for examining miR-503 inhibitor in combination with chemotherapy for treatment of glioblastoma.

## Discussion

Glioblastoma multiforme is an aggressive and incurable type of brain cancer associated with a very poor prognosis^17^. Due to its highly invasive nature, it is impossible to completely remove the whole tumor by surgical resection^18^. Over the past decade, there has been a growing interest in the functional role of microRNA in glioblastoma. Based solely on the literature^19, 20^, it appears that 256 of the most commonly dysregulated microRNAs are significantly overexpressed in glioblastoma^21^. However, few of these microRNAs, for example microRNA-21, microRNA-17 and microRNA-181^22, 23^, have been thoroughly investigated in regard to their expression or functional role. The functional properties of the majority remain largely unknown. Therefore, there is an urgent need to explore these powerful microRNAs, which could be used as potential targets in glioblastoma therapy.

In this study, we analyzed miRNA expression profiles from a previously published dataset. We found that microRNA-503 is highly upregulated in glioblastoma tissue compared with normal brain tissue, which is consistent with the report from Stefan Wuchty *et al*.^24^. MicroRNA-503 and microRNA-424 were shown to be regulated by apelin in pulmonary arterial hypertension. However, little was known about how microRNA-503 was upregulated in cancer cells, although there were several reports of its aberrant expression. Jongmin Kim *et al*. reported that miR-503 and miR-424, which are separated by 250 bp on the X chromosome, are transcribed as a single transcript. Zhang D *et al*. reported that miR-424 can be induced by TGF-β1 in normal fibroblasts. Building on these published data, we found that TGF-β1 induces microRNA-503 expression in glioblastoma cells. More elaborately, we performed a ChIP assay to show that smad2/3 binds the microRNA-503 promoter and thus enhances the level of microRNA-503. Furthermore, the core element bound by smad2/3 is highly conserved. It is well established that TGF-β1 signaling is dysregulated in glioblastoma. Thus, our data indicates that TGF-β1-microRNA-503 may play a key role in glioblastoma progression.

The role of microRNA-503 varies in different cancer tissues. In NSCLC and prostate cancer, microRNA-503 acts as tumor suppressor, however, in esophageal cancer and hepatocellular cancer, microRNA-503 act as an oncogene. In our study of glioblastoma cells, ectopic expression of microRNA-503 with mimics significantly increased cell growth, colony formation and anchorage-independent growth in soft agar. In contrast, knockdown of microRNA-503 sup pressed cell proliferation and anchorage-independent growth. Furthermore, overexpression of microRNA-503 significantly inhibited cell apoptosis. This suggests that microRNA-503 functions as an oncogene and is upregulated in glioblastoma. Moreover, our data showed that PDCD4 is a downstream target of microRNA-503. To translate our findings, we found that inhibition of endogenous microRNA-503 augments the growth inhibitory effect of temozolomide. The median effect analysis showed that microRNA-503 inhibitor and temozolomide at high concentration have a synergistic effect. These results provide a potential preclinical rationale for examining miR-503 inhibitor in combination with chemotherapy for glioblastoma treatment.

In summary, our study shows that microRNA-503 is overexpressed in glioblastoma tissues. Furthermore, TGF-β1 induces microRNA-503 at the transcriptional level by binding a core promoter element. We found that microRNA-503 increases proliferation of glioblastoma cells and inhibits apoptosis by directly targeting PDCD4. Finally, miR-503 inhibitor augments the growth inhibitory effect of temozolomide in glioblastoma cells. These results establish miR-503 as a promising molecular target for glioblastoma therapy.

## Materials and Methods

### Cell lines and reagent

U251, A172, T98G, LN-229 and 293 T were purchased from ATCC. All the cell lines were grown in Dulbecco’s Modified Eagle Medium (DMEM) supplemented with 10% FBS. The cells were passaged continuously for fewer than 6 months after receipt in our laboratory for relevant studies reported here.

### Cell transfection

Cells were transfected with 20 nM miR-503 mimics, miR-503 inhibitor or negative controls (Shanghai GenePharma) using RNAiMAX transfection reagent (Life Technologies) according to the manufacturer’s instructions. For plasmid transfections, cells were transfected with Lipofectamine 2000, according to the manufacturer’s protocol.

### Western blot

The total cell lysates were prepared with RIPA lysis buffer with complete protease inhibitor cocktail. The protein concentration was measured with the BCA Protein Assay Kit. Cell lysates were then subjected to 8% SDS-PAGE and transferred to a PVDF membrane, and probed with the indicated primary antibodies and secondary antibodies.

### Quantitative real-time PCR

Total RNA was extracted with TRIzol reagent. The RNA was subsequently treated with RNase-free DNase I. The cDNA was synthesized with BcaBEST RNA PCR kit from TaKaRa according to the manufacturer’s instructions. For detection of miR-503 expression, stem-loop RT-PCR was performed. Quantitative real-time PCR was performed by using SYBR green reagent with ABI Prism 7500 Fast detection system. Relative expression was normalized to the expression of U48 small RNA or GAPDH and measured by a comparative CT method.

### 3′ UTR luciferase reporter assay and luciferase promoter assay

For the 3′ UTR luciferase reporter assay, the miR-503 mimics or inhibitor and pmirGLO, pmirGLO-PDCD4 3′ UTR-wt, pmirGLO-PDCD4–3′ UTR-mut were cotransfected into HEK293T. Cell lysates were collected at 48 hours post-transfection. Luciferase activity was measured using the Dual-Luciferase Reporter Assay (Promega). For the luciferase promoter assay, different segments of the miR-503 promoter were produced by PCR using primers and were cloned into pGL3 vectors. The potential binding sites for TGF-b were mutanted for site-specific mutagenesis. U251 cells were transfected with pGL-miR-503-wt-set4, pGL-miR-503-mut-SBE1, pGL-miR-503-mut-SBE2, pGL-miR-503-mut-SBE3, or pGL-miR-503-mut-SBE4 and Renilla. Luciferase activity was measured using the Dual-Luciferase Reporter Assay (Promega).

### Chromatin immunoprecipitation assay

5 million U251 cells were prepared for chromatin immunoprecipitation (ChIP) using the ChIP Assay Kit (Cell Signaling Technology) according to the manufacturer’s instructions. The precipitated DNA was analyzed using PCR to amplify five 500 bp regions (set 1, set 2, set 3, set 4, set 5) of the miR-503 promoter. The PCR products were resolved by electrophoresis onto 1% agarose gel and visualized with ethidium bromide staining.

### Cell proliferation assay

For the cell proliferation assay, 5000 cells/well were seeded into 96 well plates. Cell proliferation was determined using AlamarBlue Cell Viability Assay (ThermoFisher Scientific). Data represents the mean + SD of independent experiments.

### Soft agar assay

5000 cells were suspended in a 24-well plate with soft agar and colonies were scored after 4 weeks under a dissection microscope. All experiments were performed three times in triplicate.

### Apoptosis assay

Apoptotic cells were detected by flow cytometry using the PE Annexin V apoptosis detection kit (BD Pharmingen) according to the manufacturer’s protocol.

### *In vitro* drug treatment and synergism analysis

Cells were seeded in duplicate in 96-well plates 24 hours before exposure to various concentrations of miR-503 inhibitor (The miR-503 inhibitor and temozolomide were used alone at 0.31 uM, 0.62 uM, 1.25 uM, 2.5 uM, 5 uM, 10 uM, 20 uM, 40 uM, and in combination at equipotent concentrations at the same ratios) and temozolomide in constant molar ratios. After 72 hours, Alamar Blue Assay was performed to assess viability. Median effect analysis was performed using Compusyn software. Data was combined from three independent experiments, which were each conducted in duplicate and presented as mean = SEM.

### Statistical analysis

Data were analyzed using the two-tailed Student’s t test and were shown as mean ± SE. P < 0.05 was considered statistically significant.

## Electronic supplementary material

Supplementary Information 
