# Author Correction: TGF-β1-induced miR-503 controls cell growth and apoptosis by targeting PDCD4 in glioblastoma cells

**DOI:** 10.1038/s41598-019-42588-x

**Published:** 2019-04-17

**Authors:** Pin Guo, Yanan Yu, Huanting Li, Daoxiang Zhang, Anjing Gong, Shifang Li, Wei Liu, Lei Cheng, Yongming Qiu, Weicheng Yao, Luo Li, Yugong Feng

**Affiliations:** 1 Department of Neurosurgery, the Affiliated Hospital of Qingdao University, Qingdao, China; 2 Department of Gastroenterology, the Affiliated Hospital of Qingdao University, Qingdao, China; 3 Division of Oncology, Department of Internal Medicine, Washington University School of Medicine, Saint Louis, MO 63110 USA; 4 Department of Neurosurgery, South Campus, Renji Hospital, Shanghai Jiao Tong University School of Medicine, Shanghai, China; 5 Department of Neurology, Qingdao Municipal Hospital, Qingdao University, Qingdao, China

Correction to: *Scientific Reports* 10.1038/s41598-017-11885-8, published online 14 September 2017

In the original version of this Article, all instances of “TGF-β1” were incorrectly written as “TGF-â1”.

These errors have now been corrected in the HTML and PDF versions of the Article.

