# Supplementary material for: TGF-β1-induced miR-503 controls cell growth and apoptosis by targeting PDCD4 in glioblastoma cells
Source: Sci Rep. 2017 Sep 14;7:11569. doi: 10.1038/s41598-017-11885-8 (PMC5599596; doi:10.1038/s41598-017-11885-8)
Supplement: Supplementary file 1 — Supplementary Information [file 41598_2017_11885_MOESM1_ESM.doc]

**TGF-β1-induced miR-503 controls cell growth and apoptosis by targeting PDCD4 in glioblastoma cells**

Pin Guo^1,*^, Yanan Yu^1,*^, Huanting Li^1^, Daoxiang Zhang^3^, Anjing Gong^1^, Shifang Li^1^, Wei Liu^1^, Lei Cheng^1^, Yongming Qiu^2^,Weicheng Yao^1^, Luo Li^1,4^, Yugong Feng^1,4^

1 Department of Neurosurgery, the Affiliated Hospital of Qingdao University, Qingdao, China.

* These two authors contributed equally to this article.

2 Department of Neurosurgery, South Campus, Renji Hospital, Shanghai Jiao Tong University School of Medicine, Shanghai, China.

3 Division of Oncology, Department of Internal Medicine, Washington University School of Medicine, Saint Louis, MO 63110

4 Corresponding Authors: Luo Li, Department of Neurosurgery, the Affiliated Hospital of Qingdao University, Qingdao, China; Yugong Feng, Department of Neurosurgery, the Affiliated Hospital of Qingdao University, Qingdao, China, [fengyugong001@163.com](mailto:fengyugong001@163.com).


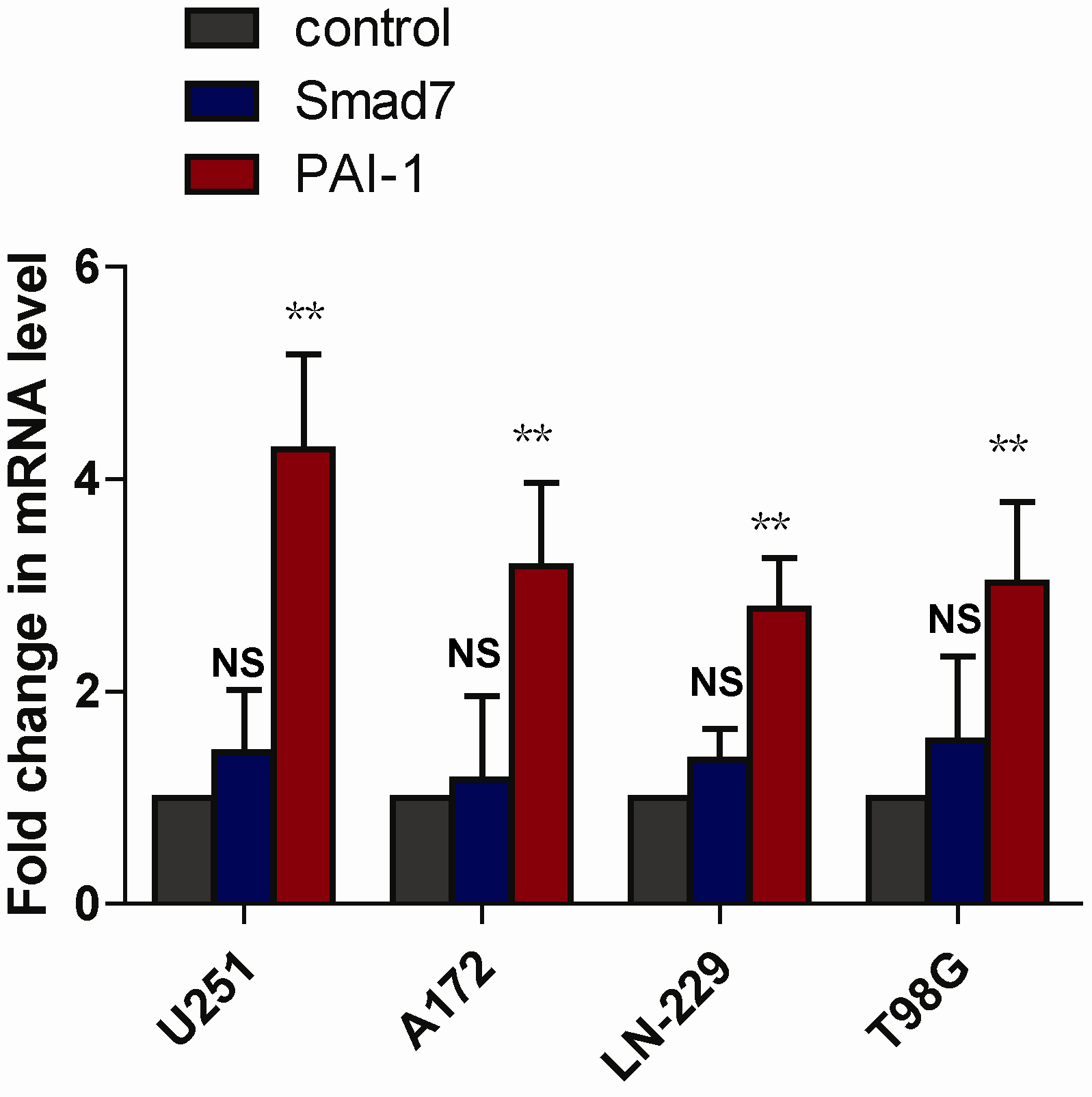


**Figure 1S TGF-β1 induces the expression of PAI-1 significantly, but not Smad7.** The PAI-1 mRNA and Smad7 mRNA were detected using quantitative PCR in cells treated with TGFβ-1 for 48h.
